# Respiratory acidosis during bronchoscopy-guided percutaneous dilatational tracheostomy: impact of ventilator settings and endotracheal tube size

**DOI:** 10.1186/s12871-019-0824-5

**Published:** 2019-08-09

**Authors:** Christian Karagiannidis, Michaela L. Merten, Leo Heunks, Stephan E. Strassmann, Simone Schäfer, Friederike Magnet, Wolfram Windisch

**Affiliations:** 1 Department of Pneumology and Critical Care Medicine, Cologne-Merheim Hospital, Kliniken der Stadt Köln GmbH, Witten/Herdecke University Hospital, Ostmerheimer Strasse 200, D-51109 Cologne, Germany; 2 Dept of Intensive Care Medicine, Amsterdam UMC, location VUmc, Amsterdam, The Netherlands

**Keywords:** Dilatational tracheostomy, Endotracheal tube, Hypercapnia, Respiratory acidosis, Transcutaneous PCO_2_

## Abstract

**Background:**

The current study investigates the effect of bronchoscopy-guided percutaneous dilatational tracheostomy (PDT) on the evolution of respiratory acidosis depending on endotracheal tube (ET) sizes. In addition, the impact of increasing tidal volumes during the intervention was investigated.

**Methods:**

Two groups of ICU-patients undergoing bronchoscopy-guided PDT with varying tidal volumes and tube sizes were consecutively investigated: 6 ml/kg (*N* = 29, mean age 57.4 ± 14.5 years) and 12 ml/kg predicted body weight (*N* = 34, mean age 59.5 ± 12.8 years).

**Results:**

The mean intervention time during all procedures was 10 ± 3 min. The combination of low tidal volumes and ETs of 7.5 mm internal diameter resulted in the most profound increase in PaCO_2_ (32.2 ± 11.6 mmHg) and decrease in pH-value (− 0.18 ± 0.05). In contrast, the combination of high tidal volumes and ETs of 8.5 mm internal diameter resulted in the least profound increase in PaCO_2_ (8.8 ± 9.0 mmHg) and decrease of pH (− 0.05 ± 0.04). The intervention-related increase in PaCO_2_ was significantly lower when using higher tidal volumes for larger ET: internal diameter 7.5, 8.0 and 8.5: *P* > 0.05, =0.006 and = 0.002, respectively. Transcutaneous PCO_2_ monitoring revealed steadily worsening hypercapnia during the intervention with a high correlation of 0.87 and a low bias of 0.7 ± 9.4 mmHg according to the Bland-Altman analysis when compared to PaCO_2_ measurements.

**Conclusions:**

Profound respiratory acidosis following bronchoscopy-guided PDT evolves in a rapid and dynamic process. Increasing the tidal volume from 6 to 12 ml/kg PBW was capable of attenuating the evolution of respiratory acidosis, but this effect was only evident when using larger ETs.

**Trial registration:**

DRKS00011004. Registered 20th September 2016.

**Electronic supplementary material:**

The online version of this article (10.1186/s12871-019-0824-5) contains supplementary material, which is available to authorized users.

## Background

Today, percutaneous dilatational tracheostomy (PDT) has become one of the most commonly used interventions in ICU medicine [1–4]. Different techniques have been developed, but guidance by video bronchoscope has been suggested to be clinically reasonable for direct visualization of tracheal puncture [5–10]. In particular, such guidance is anticipated to find the optimal puncture side, to avoid infringing of the tracheal cartilage and to secure placement of the cannula in the optimal position [11]. Thus, guidance of bronchoscopy during PDT has widely been accepted to reduce complication rates, most importantly the injury of the tracheal posterior wall [12–16]. Therefore, in Germany, 97.7% of all ICUs use bronchoscopes to guide tracheostomy [17]. However, one of the major disadvantages of bronchoscopic guidance is the partial occlusion of the endotracheal tube (ET), leading to impaired alveolar ventilation. Surprisingly, few studies have evaluated the effect of bronchoscopy during PDT on gas exchange, in particular CO_2_ retention. Here, one early study has reported a mean increase in PaCO_2_ of 24 mmHg [18, 19]. In addition, a substantial increase in PaCO_2_ has also been established during flexible bronchoscopy in stable patients using sedation practices [20].

However, PCO_2_ is frequently not monitored during PDT in clinically routine. Even though high levels of hypercapnia are suggested to be generally tolerated by patients, negative effects of hypercapnia on organ function, most importantly regulation of cerebral blood flow [20], worsening of right heart function [21, 22] and catecholamine excess in severe hypercapnia [23, 24] have also been reported. In particular, the impact of dynamic alterations with rapidly changing PCO_2_ and pH values on organ function in ICU patients with already existing organ dysfunction has yet not been fully elucidated. In this regard, there is no guideline-based recommendation regarding how to therapeutically respond to worsening alveolar ventilation resulting from the intervention. In particular, it also remains unclear whether actions such as choosing a larger ET size or increasing tidal volumes during the procedure are capable of attenuating or even avoiding the intervention-related increase in PCO_2_. This area has never been systematically investigated.

For these reasons, the present study was primarily aimed at assessing, firstly, the impact of bronchoscopy during PDT on periprocedural hypercapnic acidosis and, secondly, the impact of changing the ventilator settings in dependence of the tube size. Thereby, it was hypothesized that, first, there will be a significant increase in PaCO_2_ during PDT resulting in respiratory acidosis, and, secondly, that this increase could be attenuated by increasing the tidal volume (V_t_) during the procedure and by choosing larger ET sizes. Secondarily, it was also hypothesized that the increase in PaCO_2_ during PDT is a dynamic process, which could be further displayed by transcutaneous PCO2 monitoring (PtcCO_2_). If so, this technique would be capable of helping physicians to more safely perform PDT.

## Methods

The study was approved by the Ethics Committee of the Witten/Herdecke University (research ethics board number 101/2015 – August 2015) and registered at the German Clinical Trial Register and the WHO trial register (DRKS00011004).

### Study design

Patients with acute respiratory failure requiring invasive mechanical ventilation following intubation and planned for PDT were included into the study. Patients were eligible if informed consent could be obtained from the caregivers or legal guardians, respectively, in advance and if ventilator settings revealed a positive end expiratory pressure (PEEP)-level of ≤15 cmH_2_O and a V_t_ of 6 ml/kg predicted body weight (PBW), resulting in an arterial pH > 7.20. ET sizes had been determined clinically on individual requirements prior to the study, and these sizes were not subject to alteration during the study.

Prior to the intervention, all patients were ventilated with a fraction of inspired oxygen (FiO_2_) of 1.0 and then sedated to a Richmond Agitation Sedation Scale (RASS) of − 5, with muscle relaxants then being administered (1 mg/kg body weight rocuronium bromide). PtcCO_2_ was continuously monitored using a SenTec Digital Monitor with a sampling rate of one measurement per minute (SenTec Digital Monitoring System; SenTec AG; Ref.: 005856, MPB-Software: V05.00.15 and SMB-Software: V07.00.6; SenTec AG, Therwil, Switzerland) as described previously [21, 22]. Equilibrium measurements were established prior to intervention. A sensor was placed on the forehead of the patient at least 60 min prior to the start of the study.

**Fig. 1:**
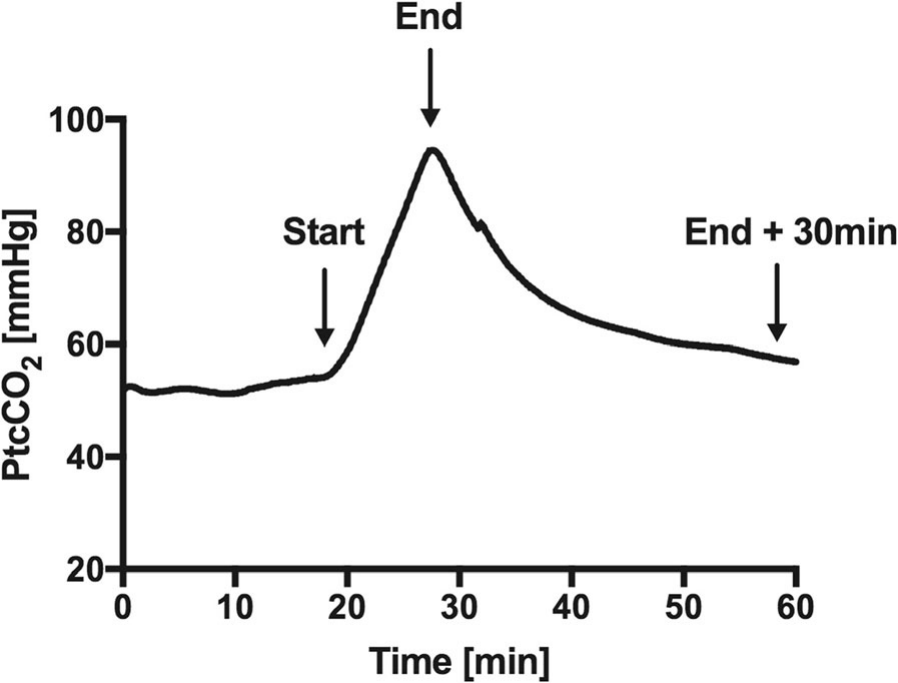
Typical example of recording transcutaneous PCO_2_ during dilatational tracheostomy (endotracheal tube size 7.5 mm ID). Arterial blood gas analysis was measured at the start, the end and the end+ 30 min, respectively

Arterial blood gas analysis was performed at the beginning of the intervention (start), at the end (end) and at 30 min after the intervention (end+ 30 min). The beginning of the intervention was defined as the time when placing the bronchoscope into the endotracheal tube. Accordingly, the end of the intervention was defined as the time when the bronchoscope was removed after having successfully placed the tracheal cannula. A typical example is provided in Fig. 1.

Two groups of patients were consecutively investigated. For both groups, pressure-controlled ventilation (Servo-I, Maquet Cardiopulmonary, Rastatt, Germany) was used throughout the entire study. Initially, ventilation was aimed at achieving a V_t_ of 6 ml/kg PBW as was done also clinically in these patients prior to the intervention (low V_t_ group). Then, a V_t_ of 12 ml/kg PBW was chosen in subsequent patients (high V_t_ group). Investigators were not blinded for the V_t_ groups. Adjustment to 12 ml/kg PBW for the purpose of the study was initiated just before the bronchoscope was inserted through the endotracheal tube. V_t_ was then reduced to 6 ml/kg PBW immediately after the bronchoscope was removed from the endotracheal tube. No further changes in ventilator settings were dictated by the study protocol. Respiratory rate, PEEP and inspiration-to-expiration ratio were maintained at a level set by the clinician prior to the procedure.

Technically, PDT was guided by video bronchoscopy (Olympus BF-Q180, maximal diameter 5.5 mm) and performed using the technique described by Ciaglia et al. (Ciaglia Blue Rhino® G2, COOK medical, Bloomington, USA) [25]. Between the start and end of the procedure, the bronchoscope was not intermittently removed due to safety reasons (possible ET displacement) and also in order to keep the duration of the procedure at a minimum. Tracheostomy was positioned in the midline of the trachea below the second to fourth tracheal ring as described previously [26]. For that purpose, a tracheal cannula with an internal diameter (ID) of 8.0 mm was preferentially chosen. All interventions were performed by an experienced intensivist or by a trainee under the direct supervision of the experienced intensivist.

### Statistical analysis

For statistical analysis, the Kruskal–Wallis one-way analysis of variance was used to compare three parameters, and the Mann-Whitney test was employed to compare 2 parameters. PaCO_2_ was compared to PtcCO_2_ at three different time points: start of the intervention, end of the intervention and 30 min after the end of the procedure. For this purpose, both correlation (spearman’s correlation) and Bland-Altman analyses were performed using GraphPad prism version 7.

## Results

Sixty-three patients were included in the study. Patient characteristics and baseline physiological data are provided in Table 1. In eight patients, tracheal rings were fractured without significant clinical impact, even after decannulation. The intervention was not interrupted or discontinued in any case.

**Table 1:** Patient’s characteristics, ventilator settings and blood gas analysis prior to tracheostomy (PBW – predicted body weight)

Main characteristics	6 ml/kg PBW group	12 ml/kg PBW group
Patient Number	*N* = 29	*N* = 34
Age [years]	57.4 ± 14.5	59.5 ± 12.8
Tracheostomy [day]	16.8 ± 8.3	16.3 ± 8.9
SAPS II	36.7 ± 10.6	36.5 ± 10.0
FiO_2_	0.42 ± 0.10	0.41 ± 11.5
P/F ratio [mmHg]	241.1 ± 196.6	211.6 ± 66.7
V_t_ [ml]	461.8 ± 102.3	462.9 ± 108.7
Breathing frequency [/min]	20.8 ± 3.4	20.2 ± 3.0
Driving Pressure [cmH_2_O]	14.6 ± 4.8	12.7 ± 3.6
PIP [cmH_2_O]	26.1 ± 5.8	24.3 ± 6.3
PEEP [cmH_2_O]	11.3 ± 2.9	11.6 ± 3.1
pH	7.37 ± 0.07	7.36 ± 0.06
PaO_2_ [mmHg]	81.8 ± 14.7	77.6 ± 17.4
PaCO_2_ [mmHg]	50.9 ± 12.9	57.0 ± 15.2

Data are presented as mean with standard deviation

### Low V_t_ group

**Fig. 2:**
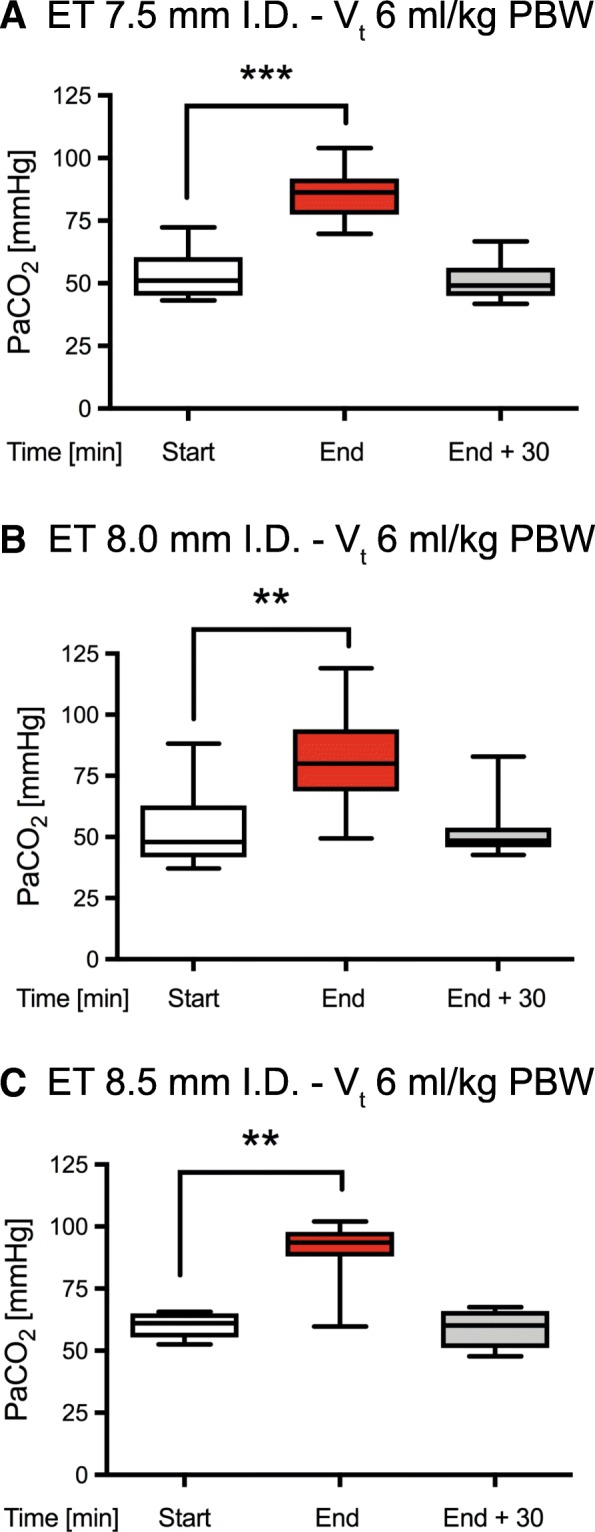
PaCO_2_ during dilatational tracheostomy. Tidal volume was set to 6 ml/kg PBW for the entire period. *p ≤ 0.05, **p ≤ 0.01 and ***p ≤ 0.001. (ET - endotracheal tube, I.D. - internal diameter). Data were analyzed according to the diameter of the tube (**a**: 7.5mm, **b**: 8.0mm and **c**: 8.5mm)

Overall, 29 patients were investigated in the low V_t_ group: 10 patients with an ET of 7.5 mm ID, 11 patients with an ET of 8.0 mm ID, and 8 patients with an ET of 8.5 mm ID, respectively. The corresponding intervention time was 12 ± 3, 11 ± 3 and 9 ± 3 min, respectively. There was a statistically significant and substantial increase in PaCO_2_ during the intervention (start to end), whereas PaCO_2_ substantially decreased following the intervention (end + 30 min) (Fig. 2). Correspondingly, pH significantly and substantially decreased and subsequently increased, respectively (Additional files 1 and 4).

### High VT group

Overall, 34 patients were investigated in the high V_t_ group: 11 patients with an ET of 7.5 mm ID, 13 patients with an ET of 8.0 mm ID and 10 patients with an ET of 8.5 mm ID, respectively. The corresponding intervention time was 10 ± 3, 10 ± 3 and 9 ± 3 min, respectively. There was a substantial increase in PaCO_2_ during the intervention (start to end), whereas PaCO_2_ substantially reduced following the intervention (end + 30 min) (Fig. 2). Correspondingly, pH markedly decreased and subsequently increased, respectively (Additional files 1 and 4). However, changes in PaCO_2_ and pH were attenuated with larger tube sizes and even did not reach statistical significance when using an ET of 8.5 mm ID.

### Comparison of low and high V_t_ groups

**Fig. 3:**
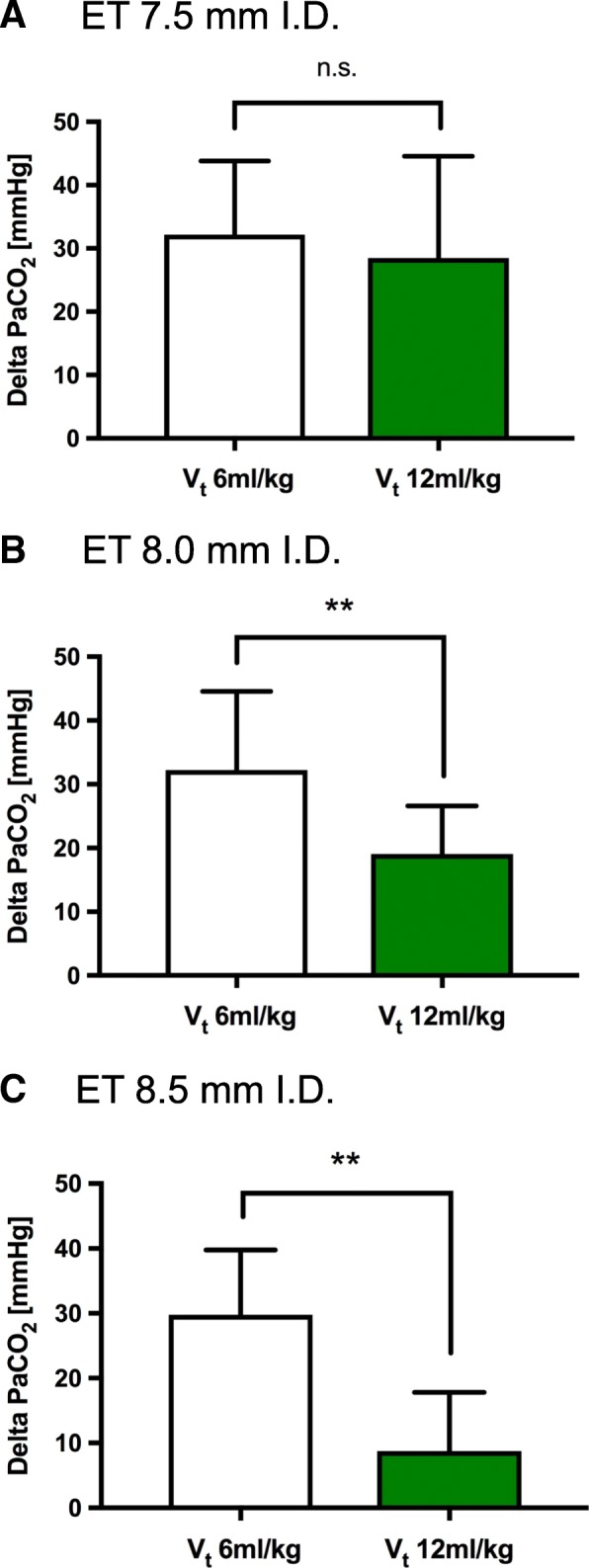
Increase of PaCO_2_ during dilatational tracheostomy. Arterial blood gas analysis was performed at the beginning and end of the procedure. Patients were ventilated with tidal volumes (V_t_) of 6 or 12 ml/kg PBW during intervention. *p ≤ 0.05 and **p ≤ 0.01. (ET - endotracheal tube, I.D. - internal diameter). Data were analyzed according to the diameter of the tube (**a**: 7.5mm, **b**: 8.0mm and **c**: 8.5mm)

**Fig. 4:**
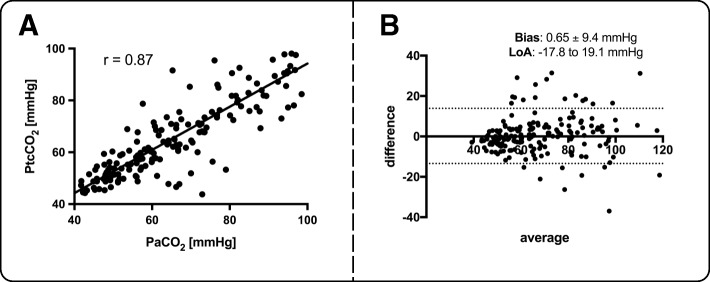
Correlation of transcutaneous PCO_2_ (PtcCO_2_) and arterial PCO_2_ (PaCO_2_) (**a**). Corresponding Bland-Altman analysis are given in (**b**). (r - spearman’s correlation; LoA - Limits of Agreement). Please note that the bias line for the Bland-Altman analysis is not visible due to the observation that the bias was close to zero

**Fig. 5:**
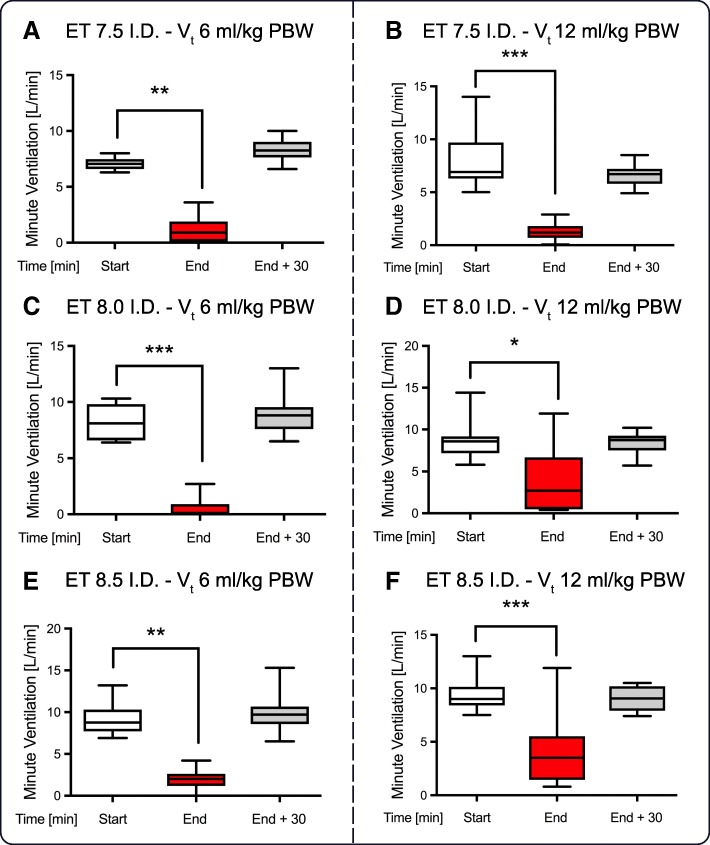
Minute ventilation during dilatational tracheostomy. Tidal volume was set to 6 ml/kg PBW (**a**, **c** and **e**) or 12 ml/kg PBW (**b**, **d** and **f**) for the entire period. *p ≤ 0.05, **p ≤ 0.01 and ***p ≤ 0.001. (ET - endotracheal tube, I.D. - internal diameter)

The increase in PaCO_2_ and the reduction in pH, respectively, were comparable with regard to different V_t_ when using an ET of 7.5 mm ID (Figs. 3 and 4, Additional files 1, 2, 3 and 4). When using an ET of 7.5 mm ID, the minute ventilation decreased during the intervention to a minimum, and this ventilation was unchanged when comparing low and high V_t_ (Fig. 5). In contrast, when using larger ET IDs, the minute ventilation during the intervention was higher when using high V_t_ compared to low V_t_. When comparing low and high V_t_, the difference in the increase in PaCO_2_ and the decrease in pH during the intervention, respectively, were more evident with higher ET IDs. Thus, respiratory acidosis occurring during the intervention could be at best attenuated when using an ET ID of 8.5 mm, while simultaneously using a V_t_ of 12 ml/kg PBW.

### Comparison of PaCO_2_ and PtcCO_2_

Hypercapnia immediately started to occur at the beginning of the intervention as assessed by PtcCO_2_ monitoring. A typical example of PtcCO_2_ monitoring during PDT is displayed in Fig. 1. PaCO_2_ and PtcCO_2_ were significantly correlated (*r* = 0.87, *p* < 0.001, Fig. 4a). The corresponding Bland-Altman analysis is displayed in Fig. 4b (Bias 0.65 ± 9.4 mmHg within the limits of agreement from − 17.8 to 19.1 mmHg).

## Discussion

The current study has demonstrated that there is a rapidly evolving respiratory acidosis during bronchoscopy-guided PDT. Thereby, the increase in PaCO_2_ is substantial and results from a reduced minute ventilation following bronchoscopy-related ET occlusion if pressure-controlled ventilation is used with established settings, aiming at lung protective ventilation. The present study has, furthermore, illustrated that an increase in V_t_ from 6 to 12 ml/kg PBW is capable of attenuating respiratory acidosis resulting from bronchoscopy-guided PDT. However, this effect was shown to be significantly dependent on ET size. Here, when using an ET of 7.5 mm ID, increasing V_t_ as described above did not result in a significant attenuation of respiratory acidosis, and this lack of attenuation is suggested to be related to the observation that the ET is sub-totally occluded by the bronchoscope when using an ET of 7.5 mm ID, thus preventing minute ventilation to be increased despite increasing inspiratory pressures aimed at achieving a V_t_ of 12 ml/kg PBW. This work also provides evidence for the impact of smaller bronchoscopes potentially improving alveolar ventilation compared to larger ones, but this area was not investigated in the present study.

As an alternative approach to ET, using a laryngeal mask is suggested to be associated with an attenuation of the increase in PCO_2_. However, there is an ongoing debate on whether laryngeal masks should be used for PDT. In this regard, a recent Cochrane analysis [27] revealed a higher probability of a failing procedure and an uncertainty of serious adverse events when using laryngeal masks. Furthermore, at least in the ICU setting used for the current study changing the airway access is at risk for even severe complications, and this should be weighted against the benefit of a less severe increase in PCO_2_.

In contrast, when using larger ET IDs, minute ventilation was shown to be increased with higher V_t_. Accordingly, respiratory acidosis occurring during bronchoscopy-guided PDT could be most successfully attenuated when combing an ET of 8.5 mm ID with a V_t_ of 12 ml/kg PBW during the intervention. In the present study, however, only pressure-controlled ventilation was used. Theoretically, volume-controlled ventilation allowing for high inspiratory pressures is suggested to provide further advantages, at least if the airway is not sub-totally occluded when using considerably low ET sizes as discussed above. However, this is clearly dependent on how alarm settings are chosen, which in this study already reached 45 cmH_2_O. Therefore, best ventilator settings aimed at avoiding PDT-related respiratory acidosis need to be established in the future. Finally, the study has also shown that PtcCO_2_ monitoring is a reliable tool for displaying the dynamic change of alveolar ventilation during bronchoscopy-guided PDT in the ICU.

The present study has some important clinical implications. Firstly, vasodilatation as caused by hypercapnia and rapidly occurring respiratory acidosis can result in an increased cerebral pressure [20], electrolyte disturbances and impairment of infection control [28, 29], whereas pulmonary vasoconstriction is prone to worsening of right and left heart function [22, 23]. Even though impairments of infection control are suggested to be of minor importance given the short intervention time, all other physiological changes related to rapidly occurring respiratory acidosis may be of particular importance for patients with predisposing conditions such as neurosurgical patients or those with multi organ failure. Thus, respiratory acidosis occurring during bronchoscopy-guided PDT as described in the present study may be harmful for ICU patients.

Secondly, clinicians should be aware that the increase of PCO_2_ during for bronchoscopy-guided PDT could be attenuated by the combination of an increased V_t_ (12 versus 6 ml/kg PBW) and a larger ET size (≥8 mm ID), but this statement is not true when only using a smaller ET size (< 8 mm ID). Theoretically, changing the ET prior to the intervention could be advantageous when solely examining the results of the current findings, but this approach is suggested to be not generally recommended for clinical routine due to the general risk of changing a tube for severely ill patients. Importantly, an increase in the V_t_ was entirely restricted to the duration of the procedure; thus, lung protective ventilation is unlikely to be abandoned. In contrast, real alveolar ventilation is still reduced as evidenced by further increased PCO_2_ values. Therefore, restricting an increase in V_t_ for the duration of the intervention is highly unlikely to cause harm for the lungs but is suggested to be capable of minimizing the occurrence of hypercapnia when using larger ET sizes as described above.

Thirdly, PCO_2_ immediately starts to increase at the beginning of the intervention and also steadily continues to increase until the intervention has finished. Accordingly, pH values consistently decrease, resulting in severe respiratory acidosis. Therefore, there is evidence to suggest that the intervention time plays a fundamental role in the occurrence of respiratory acidosis resulting from bronchoscopy-guided PDT. The duration of the intervention in the current study was in a clinically acceptable range. However, an extended duration of the intervention is prone to substantially aggravate respiratory acidosis, particularly if increasing the V_t_ is unlikely to improve alveolar ventilation in case of using smaller ET sizes, as discussed above in detail. Therefore, the duration of the intervention must be kept at a minimum *or* the bronchoscope has to be removed intermittently to allow alveolar ventilation.

Finally, the current study also provides evidence that PtcCO_2_ monitoring is a reliable and helpful tool to assess the dynamic process of worsening hypercapnia. Importantly, the bias as calculated by Bland and Altman analysis ranged between − 0.5 and 1.8 mmHg, a result which is suggested to be clinically acceptable, particularly in view of previous findings in ICU medicine [30, 31]. Thereby, PtcCO_2_ monitoring clearly provides 2 important clinical advantages. First, the trend of PCO_2_ evolution is individually accessible. Second, this technique overcomes the disadvantage of spot measurements with delayed result presentation as valid for arterial blood gas analysis. Clinically, the assessment of the dynamics in hypercapnia development may help the investigator to decide to intermittently remove the bronchoscope during the procedure to guarantee maintenance of alveolar ventilation. This area, however, was not addressed in the present study and requires further investigation also considering higher complication rates related to ET displacements and longer intervention duration.

In addition, PtcCO_2_ monitoring is also suggested to be superior over end tidal PCO_2_ monitoring because ventilation-perfusion mismatching regularly occurring in ICU patients is suggested to distort PCO_2_ measurements when using end tidal monitoring [32–34]. However, there was a significant range of the limits of agreement between arterial and transcutaneous PCO_2_ measurements in the present study, a range which was even somewhat higher than previously reported in patients receiving mechanical ventilation [32–34]. This observation may be attributed to the special clinical scenario with high individual stress levels when performing the intervention and catecholamine treatment. This drawback, however, could be overcome by relating individual arterial and transcutaneous PCO_2_ measurements prior to the intervention. Furthermore, the PCO_2_ trend over time is suggested to be at least as important as the exact PCO_2_ measurement during intervention.

There are, however, some limitations of the present study. Firstly, patients were not randomized to receive 6 or 12 ml/kg PBW. However, it is suggested that this lack of randomization did not affect the results given the clear differences between the 2 ventilatory approaches, the fact that patients were not aware of the modality chosen and the tight-fitting baseline characteristics of both groups. Secondly, the number of patients in each group defined by specific levels of V_t_ and ET size was relatively small, and also not standardized, respectively. Here, the decision to select a specific ET size was taken in the emergency situation prior to the investigation, and changing ET tubes for standardisation was considered to be unethical.

## Conclusion

In conclusion, the present study has demonstrated that hypercapnia and, consequently, profound respiratory acidosis following bronchoscopy-guided percutaneous dilatational tracheostomy evolves in a rapid and dynamic process. These changes were reportedly related to substantially reduced minute ventilation. Increasing the tidal volume from 6 to 12 ml/kg PBW was capable of attenuating the evolution of respiratory acidosis related to endotracheal tube occlusion occurring as a consequence of bronchoscopy. However, this effect was only evident when using larger endotracheal tube sizes of ≥8 mm ID.

## Additional files

Additional file 1: pH-value during dilatational tracheostomy. Tidal volume was set to 6 ml/kg PBW for the entire period. *p ≤ 0.05, **p ≤ 0.01 and ***p ≤ 0.001. (ET - endotracheal tube, I.D. - internal diameter). (PDF 1669 kb)

Additional file 2: Decrease of pH-value during dilatational tracheostomy. Arterial blood gas analysis was performed at the beginning and end of the procedure. Patients were ventilated with tidal volumes (V_t_) of 6 or 12 ml/kg PBW during intervention. *p ≤ 0.05 and **p ≤ 0.01. (ET - endotracheal tube, I.D. - internal diameter). (PDF 1075 kb)

Additional file 3: Changes in PaCO_2_ during bronchoscopy-guided percutaneous dilatational tracheostomy: 6 versus 12 ml/kg PBW. (DOCX 16 kb)

Additional file 4: Changes in pH during bronchoscopy-guided percutaneous dilatational tracheostomy: 6 versus 12 ml/kg PBW. (DOCX 16 kb)

## Data Availability

All data generated or analysed during this study are included in this published article.

## Abbreviations

ET: Endotracheal tube
FiO_2_: Inspiratory fraction of oxygen
I.D: Internal diameter
ICU: Intensive care unit
PaCO_2_: Arterial partial pressure of carbon dioxide
PBW: Predicted body weight
PCO_2_: Partial pressure of carbon dioxide
PDT: Percutaneous dilatational tracheostomy
PtcCO_2_: Transcutaneous partial pressure of carbon dioxide
RASS: Richmond Agitation Sedation Scale
V_t_: Tidal volume
