# Supplementary figures and images for: Respiratory acidosis during bronchoscopy-guided percutaneous dilatational tracheostomy: impact of ventilator settings and endotracheal tube size
Source: BMC Anesthesiol. 2019 Aug 9;19:147. doi: 10.1186/s12871-019-0824-5 (PMC6689167; doi:10.1186/s12871-019-0824-5)

**A** ET 7.5 mm I.D. -  $V_t$  6 ml/kg PBW

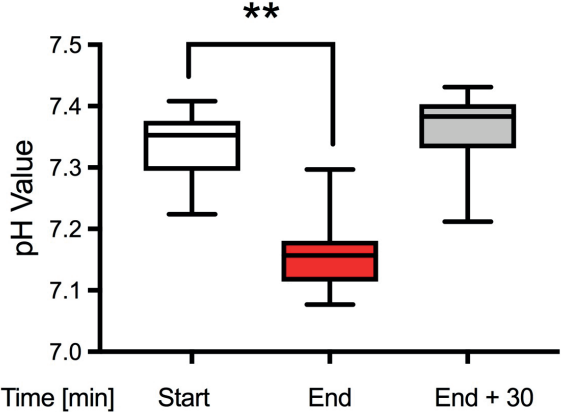

**B** ET 8.0 mm I.D. -  $V_t$  6 ml/kg PBW

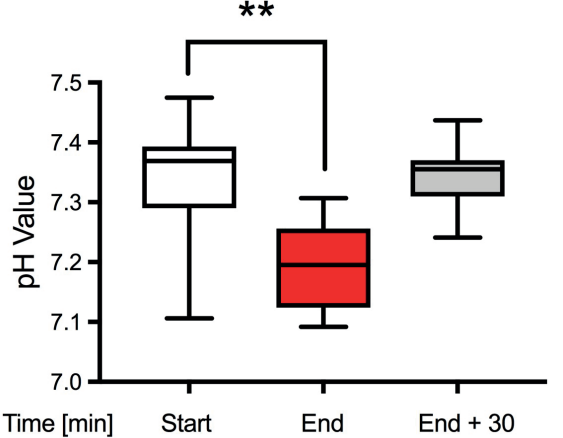

**C** ET 8.5 mm I.D. -  $V_t$  6 ml/kg PBW

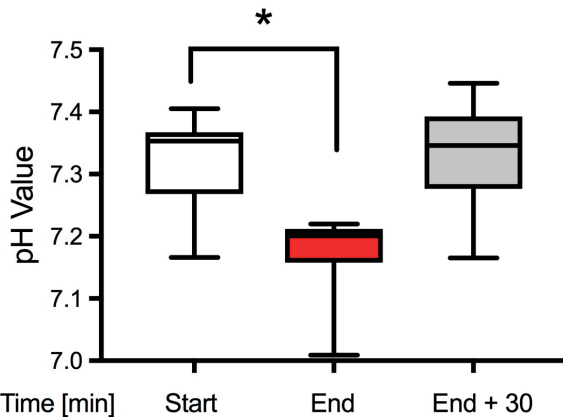

Supplement: Supplementary file 1 — pH-value during dilatational tracheostomy. Tidal volume was set to 6 ml/kg PBW for the entire period. *p ≤ 0.05, **p ≤ 0.01 and ***p ≤ 0.001. (ET - endotracheal tube, I.D. - internal diameter). (PDF 1669 kb) [file 12871_2019_824_MOESM1_ESM.pdf]

**A** ET 7.5 mm I.D.

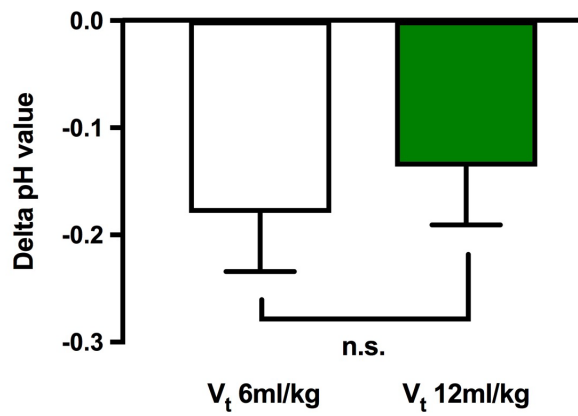

**B** ET 8.0 mm I.D.

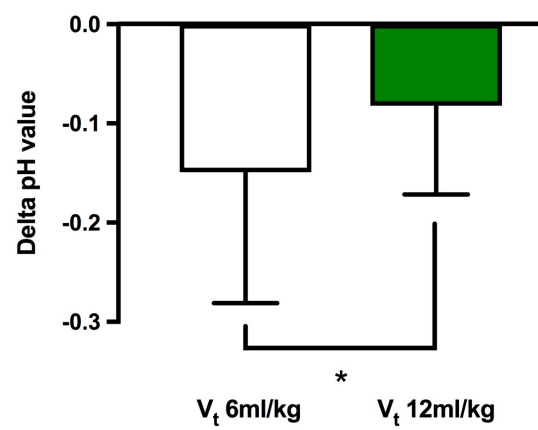

**C** ET 8.5 mm I.D.

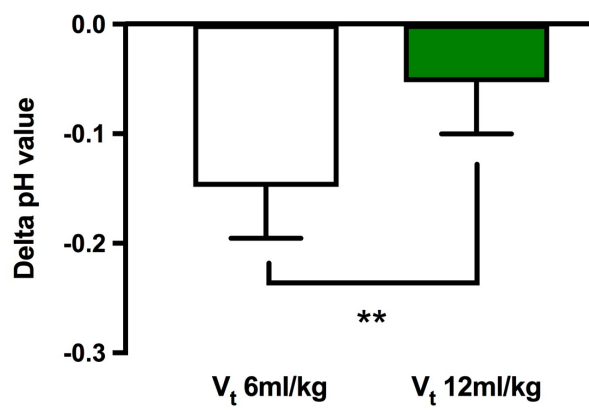

Supplement: Supplementary file 2 — Decrease of pH-value during dilatational tracheostomy. Arterial blood gas analysis was performed at the beginning and end of the procedure. Patients were ventilated with tidal volumes (Vt) of 6 or 12 ml/kg PBW during intervention. *p ≤ 0.05 and **p ≤ 0.01. (ET - endotracheal tube, I.D. - internal diameter). (PDF 1075 kb) [file 12871_2019_824_MOESM2_ESM.pdf]
