# Supplementary material for: Respiratory acidosis during bronchoscopy-guided percutaneous dilatational tracheostomy: impact of ventilator settings and endotracheal tube size
Source: BMC Anesthesiol. 2019 Aug 9;19:147. doi: 10.1186/s12871-019-0824-5 (PMC6689167; doi:10.1186/s12871-019-0824-5)
Supplement: Supplementary file 3 — Changes in PaCO2 during bronchoscopy-guided percutaneous dilatational tracheostomy: 6 versus 12 ml/kg PBW. (DOCX 16 kb) [file 12871_2019_824_MOESM3_ESM.docx]

**Additional File 3**

**Changes in PaCO_2_ during bronchoscopy-guided percutaneous dilatational tracheostomy**: 6 versus 12 ml/kg PBW

| Tube ID [mm] | Delta PaCO_2_ [mmHg]  6 ml/kg PBW | Delta PaCO_2_ [mmHg]  12 ml/kg PBW | P-value |
| --- | --- | --- | --- |
| 7.5 | 32.2 ± 11.6 | 28.5 ± 16.1 | p>0.05 |
| 8.0 | 32.2 ± 12.3 | 19.1 ± 7.5 | p=0.006 |
| 8.5 | 29.8 ± 10.0 | 8.8 ± 9.0 | p=0.002 |

PBW - predicted body weight
