# Supplementary material for: Respiratory acidosis during bronchoscopy-guided percutaneous dilatational tracheostomy: impact of ventilator settings and endotracheal tube size
Source: BMC Anesthesiol. 2019 Aug 9;19:147. doi: 10.1186/s12871-019-0824-5 (PMC6689167; doi:10.1186/s12871-019-0824-5)
Supplement: Supplementary file 4 — Changes in pH during bronchoscopy-guided percutaneous dilatational tracheostomy: 6 versus 12 ml/kg PBW. (DOCX 16 kb) [file 12871_2019_824_MOESM4_ESM.docx]

**Additional File 4**

**Changes in pH during bronchoscopy-guided percutaneous dilatational tracheostomy: 6 versus 12 ml/kg PBW**

| Tube ID [mm] | Delta pH value  6 ml/kg PBW | Delta pH value  12 ml/kg PBW | P-value |
| --- | --- | --- | --- |
| 7.5 | -0.18 ± 0.05 | -0.14 ± 0.05 | p>0.05 |
| 8.0 | -0.15 ± 0.13 | -0.08 ± 0.09 | P=0.017 |
| 8.5 | -0.15 ± 0.05 | -0.05 ± 0.04 | p=0.001 |

PBW - predicted body weight
